# Continuous monitoring of sleep‐disordered breathing with pacemakers: Indexes for risk stratification of atrial fibrillation and risk of stroke

**DOI:** 10.1002/clc.23489

**Published:** 2020-11-12

**Authors:** Andrea Mazza, Maria Grazia Bendini, Massimo Leggio, Raffaele De Cristofaro, Sergio Valsecchi, Giuseppe Boriani

**Affiliations:** ^1^ Cardiology Division S. Maria della Stella Hospital Orvieto Italy; ^2^ Department of Medicine and Rehabilitation, Cardiac Rehabilitation Operative Unit, S. Filippo Neri Hospital Rome Italy; ^3^ Rhythm Management Division, Boston Scientific Milan Italy; ^4^ Cardiology Division, Department of Biomedical, Metabolic and Neural Sciences University of Modena and Reggio Emilia Modena Italy

**Keywords:** atrial fibrillation, pacemaker, sleep apnea, stroke

## Abstract

**Background:**

Sleep apnea (SA) is a risk factor for atrial fibrillation (AF). Advanced pacemakers are now able to calculate indexes of SA severity.

**Hypothesis:**

We investigated the changes in pacemaker‐measured indexes of SA, we assessed their predictive value for AF occurrence and the associated risk of stroke and death at long‐term.

**Methods:**

We enrolled 439 recipients of a pacemaker endowed with an algorithm for the calculation of a Respiratory Disturbance Index (RDI). The RDI variability was measured over the first 12 months after implantation, as well as its potential association with the occurrence of AF, defined as device‐detected cumulative AF burden ≥6 hoursours in a day.

**Results:**

The individual RDI mean was 30 ± 18 episodes/h, and the RDI maximum was 59 ± 21 episodes/h. RDI ≥30 episodes/h was detected in 351 (80%) patients during at least one night. The proportion of nights with RDI ≥30 episodes/h was 14% (2%‐36%). AF ≥6 hours was detected in 129 (29%) patients during the first 12 months. The risk of AF was higher in patients with RDI maximum ≥63 episodes/h (HR:1.74; 95%CI: 1.22‐2.48; *P* = .001) and with RDI mean ≥ 46 episodes/h (HR:1.63; 95%CI: 1.03‐2.57; *P* = .014). The risk of all‐cause death or stroke was higher in patients with AF burden ≥6 hours (HR:1.75; 95%CI: 1.06‐2.86; *P* = .016). Moreover, among patients with no previous history of AF the risk was higher in those with RDI maximum ≥63 episodes/h (HR:1.96; 95%CI: 1.06‐3.63; *P* = .031).

**Conclusions:**

Pacemaker‐detected SA showed a considerable variability during follow‐up. We confirmed the association between RDI and higher risk of AF, and we observed an association between higher RDI maximum and all‐cause death or stroke among patients with no previous history of AF.

## 1 INTRODUCTION

Sleep apnea (SA) is a well‐known risk factor associated with increased incidence of cardiovascular morbidity, including atrial fibrillation (AF),^1^ hypertension,^2^ heart failure, and coronary artery disease.^3^ SA has also been associated with a 3‐fold increase in the risk of all‐cause mortality at long term.^4^ Moreover, in patients with sleep‐disordered breathing, a doubled to tripled risk of stroke has been observed independently on other risk factors,^5^, ^6^, ^7^ as well as an increased risk of all‐cause mortality.^8^ This association was stronger in males,^9^ and was observed also in patients with AF already treated with oral anticoagulants.^10^ SA has a prevalence of about 60% in patients implanted with pacemakers according to conventional indications.^11^ Recently, automated pacemaker algorithms have been developed to calculate a Respiratory Disturbance Index (RDI) that well correlates with the results of standard polysomnography,^12^, ^13^ and that showed a good performance in identifying patients at higher risk of new‐onset AF over the mid‐term.^14^, ^15^ The availability of these algorithms offers the opportunity to monitor sleep‐disordered breathing in the long run.

The aims of this study were to investigate the changes in sleep‐disordered breathing severity, to calculate pacemaker‐derived indexes of SA severity, to assess their predictive value for AF occurrence over 1‐year follow‐up and the associated risk of stroke and death at long‐term.

## 2 METHODS

### 2.1 Patient selection, pacemaker implantation, and follow‐up

We enrolled all consecutive adult patients in whom a pacemaker had been implanted from October 2013 to March 2019 at the Santa Maria della Stella Hospital in Orvieto, Italy. Patients were required to have standard indications for dual‐chamber pacing. Patients with evidence of systolic dysfunction (left ventricular ejection fraction ≤35%) or a prior diagnosis of heart failure were excluded from the analysis. The study was approved by the Local Ethics Committee and informed consent was obtained from all patients. Devices and pacing leads were implanted by means of standard techniques. Baseline evaluation included demographics and medical history, clinical examination, 12‐lead ECG, and echocardiographic evaluation. In the analysis, patients were considered to have history of AF if clinical AF was diagnosed before or at the time of implantation. In accordance with the ESC Guidelines for the diagnosis and management of AF,^16^ this required documentation through standard 12‐lead ECG recording or a single‐lead ECG tracing of ≥30s. Optimization of pacing parameters and pharmacological treatments was based on clinical evaluation by the attending physicians. During follow‐up, patients returned for clinic visits at 1 week, 1 month, and every 3 months thereafter. At each scheduled or unscheduled visit, the pacemaker was interrogated and stored data were retrieved.

### 2.2 Device characteristics, SA detection, and endpoints

Commercially available pacemakers and transvenous leads were used in this study. Pacemakers were equipped with the ApneaScan diagnostic feature (Boston Scientific Inc., Natick, MA, USA). This feature continuously counts respiratory acts by measuring thoracic impedance between the lead and the pacemaker can. At night, the algorithm automatically detects apnea/hypopnea events (longer than 10s) by measuring reductions in tidal volume. The Respiratory Disturbance Index (RDI) is the average number of events per hour throughout the night.^12^ SA was defined as severe if the pacemaker‐measured RDI was ≥30 episodes/h.^12^, ^17^ The night‐to‐night variability of RDI values was investigated by measuring the following indexes in each patient: the average RDI value (RDI mean), the maximum value (RDI max), the proportion of nights with RDI ≥30 episodes/h (RDI burden). The RDI variability was measured over the first 12 months after implantation, as well as its potential association with the occurrence of AF in each patient. The incidence and duration of AF were derived from device data, which comprise the total time spent by the patient in AF on each day of the follow‐up period. Patients were considered to have experienced AF episodes if the device detected a cumulative AF duration greater than or equal to 6 hours in a day, in agreement with previous studies.^18^, ^19^ The patient outcome was assessed over the entire follow‐up by measuring the combined endpoint of all‐cause death or stroke. Stroke was defined as rapidly developing signs of focal (or global) disturbance of cerebral function lasting more than 24 hours with no apparent nonvascular cause.^20^ The diagnosis of ischemic stroke was established by a neurologist after standard diagnostic tests including brain imaging. The prognostic value of pacemaker‐detected sleep‐disordered breathing was tested by measuring the association between RDI parameters and the combined endpoint.

### 2.3 Statistical analysis

Continuous data were expressed as mean ± SD or median and interquartile range (25th‐75th percentile). Categorical data were expressed as percentages. Event rates were summarized by constructing Kaplan‐Meier curves, and the distributions of the groups were compared by means of a log‐rank test. Cox proportional hazards models were used to determine the association between covariates and the occurrence of AF during the first 12 months after implantation and to estimate the hazard ratios (HRs) and the 95% confidence intervals (CIs) of an AF event. All variables associated to a *P* value <.05 on univariate analysis were entered into the multivariate regression analysis. A *P* value <.05 was considered significant for all tests. A receiver operating characteristic (ROC) curve analysis was conducted to assess the performance of the RDI measures as predictors for AF, and we regarded the value resulting in the maximum product of sensitivity and specificity on the curve as the optimal cutoff. All statistical analyses were performed by means of the STATISTICA software, version 7.1 (StatSoft, Inc.).

## 3 RESULTS

### 3.1 Study population

From October 2013 to March 2019, a total of 439 consecutive patients with a standard indication for permanent pacing underwent dual‐chamber pacemaker implantation in our center. Table 1 shows baseline clinical variables. The pacemaker was implanted for atrioventricular block in 173 cases (40%), sinus node disease in 229 (52%), and carotid sinus syndrome in 37 (8%). One hundred sixty‐three (37%) patients presented with a history of AF. Oral anticoagulant therapy was used by 134 (31%) patients on hospital discharge and by 166 (38%) at the time of the last follow‐up visit.

**TABLE 1:** Demographics and baseline clinical parameters

Parameter	N = 439
Male gender, n (%)	251 (57)
Age, years	78 ± 8
Body mass index (kg/m2)	27 ± 4
Ejection fraction, %	58 ± 7
Left atrial diameter, mm	42 ± 5
NYHA class NYHA I, n (%) NYHA II, n (%) NYHA III, n (%)	262 (60) 168 (38) 9 (2)
Coronary artery disease, n (%)	61 (14)
Hypertension, n (%)	344 (78)
Diabetes, n (%)	113 (26)
Chronic obstructive pulmonary disease, n (%)	42 (10)
Chronic kidney disease, n (%)	85 (19)
Peripheral arterial disease, n (%)	57 (13)
CHADS_2_ score	1.9 ± 0.9
CHA_2_DS_2_‐VASc score	3.2 ± 1.3
History of atrial fibrillation, n (%)	163 (37)
Beta‐blockers use, n (%)	114 (26%)
Amiodarone use, n (%)	74 (17%)
Oral anticoagulant therapy use, n (%)	134 (31%)

### 3.2 Sleep‐disordered breathing during follow‐up

**FIGURE 1:**
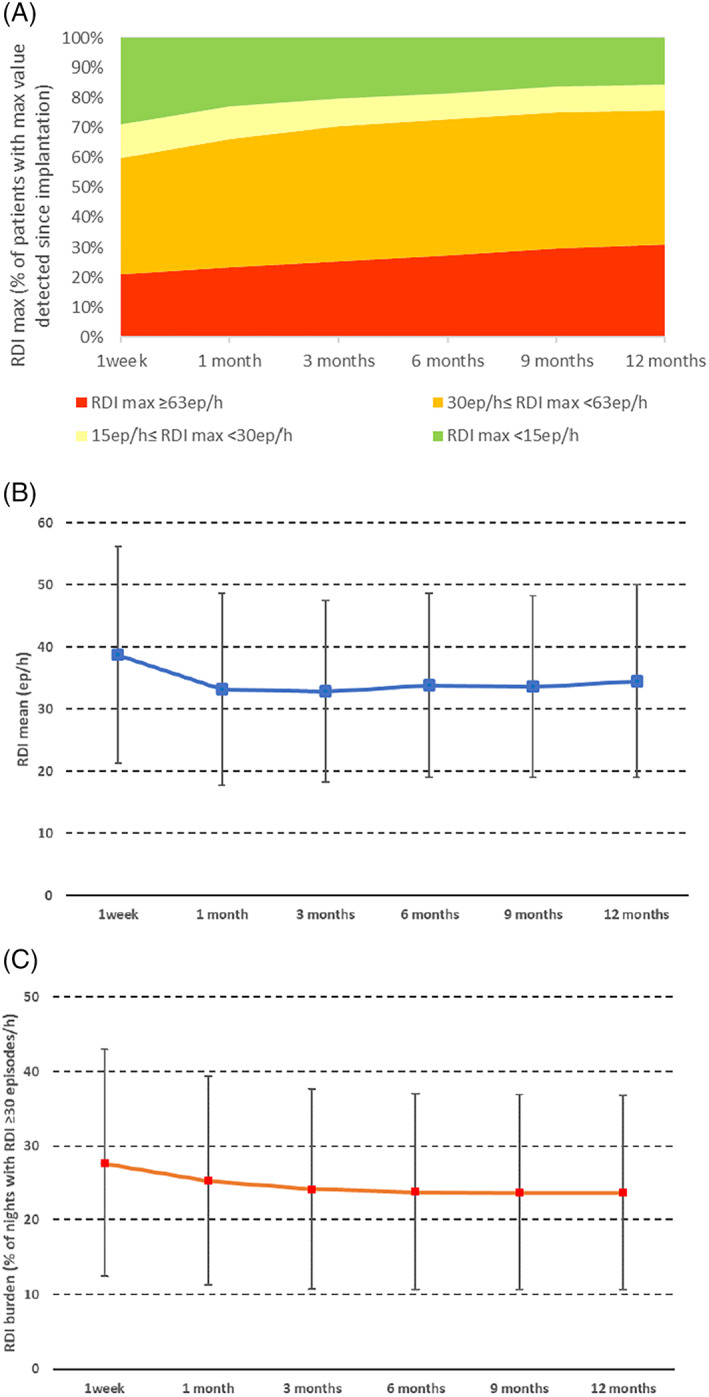
Time to reach the maximum RDI value during the first 12 months after implantation, that is, proportion of patients with RDI max value <15ep/h, <30ep/h, <63ep/h, ≥63ep/h (***Panel A***). RDI mean values calculated at different time points during follow‐up (from implantation to first week, 1 month, 3 months, 6 months, 12 months) (***Panel B***). RDI burden values calculated at different time points during follow‐up (from implantation to first week, 1 month, 3 months, 6 months, 12 months) (***Panel C***)

During the 12‐months post‐implantation period, the individual average RDI value (RDI mean) was 30 ± 18 episodes/h, and the maximum value (RDI max) was 59 ± 21 episodes/h in the study population. The individual mean night‐to‐night RDI coefficient of variation was 34 ± 17%, with an absolute SD of 11 ± 6 episodes/h for each patient. RDI ≥30 episodes/h was detected in 351 (80%) patients during at least one night. The individual median proportion of nights with RDI ≥30 episodes/h (RDI burden) was 14% (25th ‐ 75th percentile: 2% ‐ 36%). The proportion of patients achieving RDI maximum values ≥30episodes/h slightly increased from the first observation after implantation to the 12‐month visit (Figure 1). Despite a wide variability, the assessment of RDI mean value and RDI burden value seemed not affected by the length of the observation period (Figure 1).

### 3.3 Occurrence of AF and association with sleep‐disordered breathing

**FIGURE 2:**
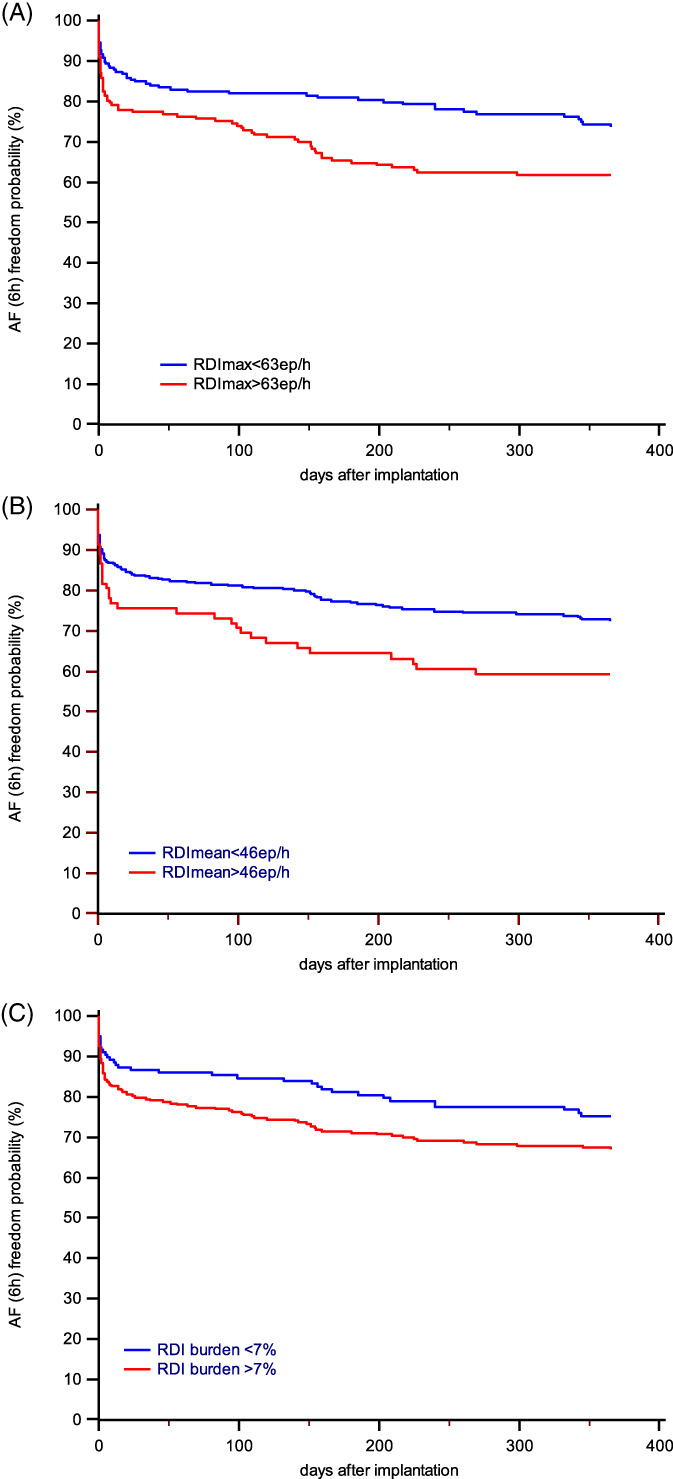
Kaplan–Meier estimates of time to AF (≥6 hours in a day), stratified by: RDI max value (***Panel A*** ‐ log‐rank test, HR: 1.74; 95%CI: 1.22‐2.48; *P* = .001), RDI mean value (***Panel B*** ‐ log‐rank test, HR: 1.63; 95%CI: 1.03‐2.57; *P* = .014), RDI burden value **(*Panel C*** ‐ log‐rank test, HR: 1.44; 95%CI: 1.01‐2.06; *P* = .057)

AF of at least 6 hours was detected in 129 (29%) patients during the first 12 months after implantation. Specifically, AF (≥6 h) occurred in 49 (18%) of the 276 patients with no history of previous AF. In patients with AF, the RDI max value was 58 ± 25 episodes/h (vs 49 ± 29 episodes/h in patients with no AF or AF <6 hours, *P* = .001), the RDI mean value was 32 ± 18 episodes/h (vs 30 ± 19 episodes/h in patients with no AF or AF <6 hours, *P* = 0.156), the RDI burden was 14% (25th‐75th percentile: 2%‐33%) (vs 14% [25th‐75th percentile: 0%‐36%] in patients with no AF or AF <6 hours, *P* = .774). Based on the ROC curve analysis, the values that maximized sensitivity and specificity for the prediction of AF were RDI max ≥63 episodes/h, RDI mean ≥ 46 episodes/h, and RDI burden ≥7%. The risk of AF was higher in patients with more severe sleep‐disordered breathing according to the three criteria (although not significantly higher for patients with RDI burden ≥7%). Figure 2 shows the Kaplan‐Meier event‐free curves regarding AF (≥6 hours in a day). On univariate analysis (Table 2), the RDI parameters that showed a significant association with AF occurrence during follow‐up were RDI max and RDI mean. Both parameters were confirmed as independent predictors of AF on multivariate analysis.

**TABLE 2:** Univariate and multivariate analysis of baseline factors associated with AF occurrence

	Univariate analysis				Multivariate analysis (1)			Multivariate analysis (2)		
	HR	95% CI	*P*		HR	95% CI	*P*	HR	95% CI	*P*
Male gender	1.02	0.72‐1.44	.923		—	—	—	—	—	—
Age	1.00	0.98‐1.02	.821		—	—	—	—	—	—
Ejection fraction	0.99	0.97‐1.02	.728		—	—	—	—	—	—
Left atrial diameter	1.06	1.03‐1.10	<.001		1.04	1.00‐1.07	.043	1.03	0.99‐1.06	.133
NYHA Class	0.90	0.65‐1.26	.557		—	—	—	—	—	—
Body mass index	1.01	0.97‐1.06	.545		—	—	—	—	—	—
History of AF	3.68	2.57‐5.24	<.001		3.45	2.34‐5.07	<.001	3.67	2.49‐5.40	<.001
Coronary artery disease	1.15	0.72‐1.86	.556		—	—	—	—	—	—
Hypertension	1.55	0.96‐2.49	.072		—	—	—	—	—	—
Diabetes	0.91	0.69‐1.24	.273		—	—	—	—	—	—
COPD	1.06	0.60‐1.88	.838		—	—	—	—	—	—
Chronic kidney disease	0.98	0.63‐1.52	.931		—	—	—	—	—	—
Peripheral arterial disease	0.92	0.55‐1.55	.761		—	—	—	—	—	—
CHADS_2_ score	1.04	0.86‐1.27	.674		—	—	—	—	—	—
CHA_2_DS_2_‐VASc score	0.98	0.86‐1.12	.767		—	—	—	—	—	—
RDI max ≥63 ep/h	1.74	1.23‐2.45	.002		1.83	1.29‐2.60	<.001	—	—	—
RDI mean ≥ 46 ep/h	1.63	1.10‐2.42	.015		—	—	—	1.78	1.19‐2.64	.005
RDI burden ≥7%	1.44	0.99‐2.11	.061		—	—	—	—	—	—
Antiarrhythmic medications	1.55	1.03‐2.34	.038		0.86	0.57‐1.33	.494	0.81	0.52‐1.25	.340

Abbreviations: AF, atrial fibrillation; COPD, chronic obstructive pulmonary disease; NYHA, New York Heart Association.

### 3.4 Clinical events over follow‐up

During a median follow‐up of 32 (25th‐75th percentile: 16‐54) months, 52 patients died. Strokes were reported in 22 patients. The risk of the combined endpoint of all‐cause death or stroke was higher in patients with pacemaker‐diagnosed AF (daily AF burden ≥6 hours) (log‐rank test, HR: 1.75; 95%CI: 1.06‐2.86; *P* = .016).

**FIGURE 3:**
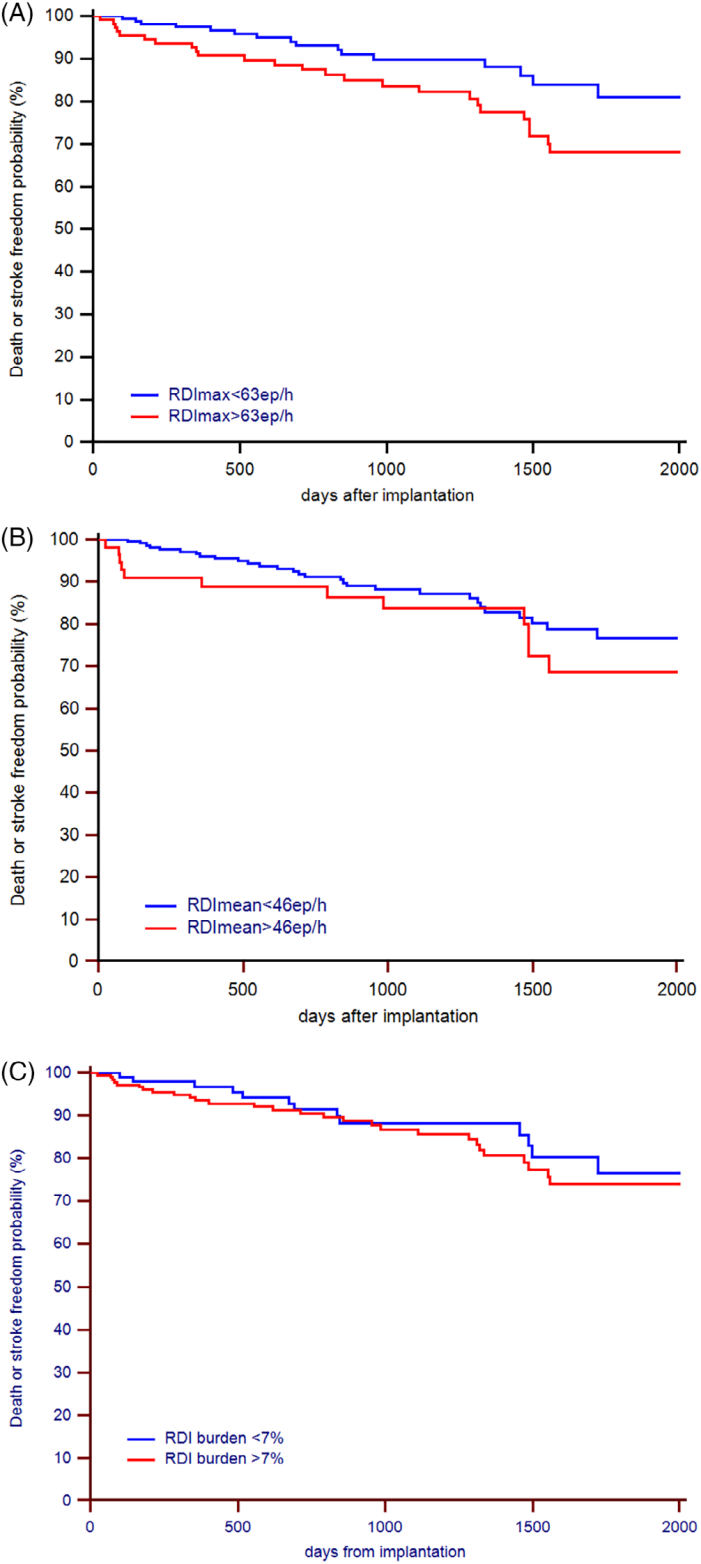
Kaplan–Meier estimates of time to all‐cause death or stroke among patients with no previous history of AF, stratified by: RDI max value (*Panel A* ‐ log‐rank test, HR: 1.96; 95%CI: 1.06‐3.64; *P* = .031), RDI mean value (*Panel B* ‐ log‐rank test, HR: 1.50; 95%CI: 0.71‐3.14; *P* = .237), RDI burden value (*Panel C* ‐ log‐rank test, HR: 1.24; 95%CI: 0.66‐2.34; *P* = .524)

The risk of death or stroke was non‐significantly higher in patients with RDI max ≥63 episodes/h (log‐rank test, HR: 1.23; 95%CI: 0.78‐1.95; *P* = .375) in the overall population, while it was significantly higher (log‐rank test, HR: 1.96; 95%CI: 1.06‐3.63; *P* = .031) among patients with no previous history of AF. Figure 3 shows the Kaplan–Meier analysis of the time to all‐cause death or stroke, according to the three RDI criteria.

## 4 DISCUSSION

In the present study, pacemaker‐detected SA showed a considerable variability during follow‐up. This large variability seems to confirm that, as previously suggested,^21^ a single overnight sleep study may not be representative of SA severity and may result in misclassification of severe SA. In our study group the incidence of severe SA was high, when continuously monitored over 12 months after pacemaker implantation. Indeed, RDI ≥30 episodes/h was detected in 80% patients during at least one night. This value is extremely higher than that reported in a previous study aimed at determining the prevalence of SA in pacemaker patients by means of standard polysomnography.^11^ Indeed, although the prevalence of undiagnosed SA was judged excessively high by the authors, it was reported that 59% of patients had an apnea‐hypopnea index ≥10 episodes/h and 21% had a severe SA (apnea‐hypopnea index ≥30 episodes/h). Despite the great night‐to‐night variability, we have seen that the proportion of patients classified according to the RDI maximum value did not change significantly in the long‐term. Similarly, the RDI mean and burden values were not affected by the length of the observation period in patients implanted with pacemakers according to conventional indications. This means that, although continuous monitoring might be helpful for removing the noise introduced by the night‐to‐night variability, substantial changes should not be expected in the classification of SA severity during follow‐up. Perhaps, this result might be different with different clinical profiles. Indeed, in heart failure patients implanted with defibrillators a worsening in SA severity might be observed with the progression of the disease or an improvement might follow effective cardiac resynchronization therapy.^22^

Previous studies have shown that SA, as detected by conventional polysomnography, was more prevalent in patients with clinical AF.^1^, ^23^ Multiple mechanisms have been proposed to explain the link between SA and AF, such as the apnea‐induced hypoxemia,^24^ the diastolic dysfunction induced by the cardiac wall stress generated by the attempted inspirations,^25^ the SA‐induced systemic inflammation,^26^ the autonomic imbalance that has been seen to occur during SA.^27^ Atrial fibrillation is common in patients with pacemakers,^28^ and devices frequently detect silent episodes in patients without a clinical history of AF.^29^ Our findings that severe SA was independently predictive of AF occurrence extends to the specific field of device‐detected subclinical AF, and confirm previous smaller studies.^14^, ^30^, ^31^ Our results suggest that device‐based RDI may be helpful in stratifying pacemaker patients for AF risk, by reviewing these automated diagnostic data during routine in‐office or remote interrogation. Indeed, the risk of AF was almost doubled in patients with SA diagnosed by the device during follow‐up.

The optimal RDI values for the prediction of AF with pacemaker, that is, RDI max ≥63 episodes/h and RDI mean ≥ 46 episodes/h, were significantly higher than the value of apnea‐hypopnea index (≥30 episodes/h) commonly adopted to diagnose severe SA with standard polysomnography.^12^, ^17^ This result is consistent with previous findings on the agreement between AHI and RDI values recorded during the sleep‐study night.^32^ Indeed, the RDI value was shown to be affected by a positive bias of 11 episodes/h. In a previous pacemaker study,^31^ the nights with the highest pacemaker‐detected SA severity seemed to confer an increased risk of having at least 1 hour of AF the same day compared to nights with the lowest SA severity. This seemed to suggest that a measure like the proportion of nights with high RDI (SDB burden), rather than a categorical diagnosis of SA per se, may be a more useful metric to determine SA severity in the management of concomitant AF. However, in our analysis RDI max and RDI mean were associated with AF occurrence during follow‐up with comparable predictive power, while RDI burden seemed a weaker predictor.

The capability of implanted cardiac devices to monitor the atrial rhythm is an opportunity to stratify patients for the risk of ischemic stroke, and may constitute an effective tool for ensuring correct antithrombotic treatment.^19^ It has previously been found that a maximum daily AF burden of 6 hours implies a 17% increase in the risk of stroke.^19^ The present results seem to confirm this finding, indeed in our patients with pacemaker‐diagnosed AF (daily AF burden ≥6 hours) the risk of all‐cause death or stroke was higher during a median follow‐up of 32 months. Interestingly, we also observed an association between higher RDI max values and the combined end‐point of all‐cause death or stroke among patients with no previous history of AF.

Confirming the observed link between AF, SA, and increased risk of stroke may have implications for targeted therapeutic strategies. Indeed, appropriate treatment with continuous positive airway pressure in obstructive SA patients has been associated with lower recurrence of AF.^24^ Although this effect was not confirmed in a more recent study,^1^ other interventions, such as treatments targeting obesity, could have a role in preventing or treating AF, and finally reducing the risk of stroke.

### 4.1 Limitations

The main limitation of the present study is the observational design of the analysis. Indeed, some variability in the selection or management of patients during the inclusion period may have influenced the results. However, the study was carried out in a single center, the operators in charge of patient selection, device implantation and clinical management did not change during the study period, and all the patients included were consecutive. Although the device measured the RDI during the entire observation period of each patient, for the sake of simplicity we only analyzed the RDI variability and its association with AF occurrence during the 12‐months post‐implantation period. Therefore, we may have underestimated the number of patients with severe SA episodes over the long‐term follow‐up, with a possible impact on the analysis of the association between SA and clinical events. Moreover, the algorithm for SA detection does not distinguish between obstructive SA and central SA. Nonetheless, central SA is most commonly present in heart failure patients with systolic dysfunction, who were not included in the present analysis. In addition, in our analysis patients were considered to have experienced AF if the device detected a cumulative daily AF burden was ≥6 hours. This threshold of AF burden has been used in other previous studies.^33^, ^34^ By using this threshold of AF burden we were therefore not able to distinguish between patients with shorter AF duration and those with no AF. In agreement with previous analyses,^18^, ^19^, ^34^ the 6 hours cutoff was chosen as an approximation of the 5.5 hours value identified in one of the studies evaluating the association between pacemaker‐detected AF and thromboembolic risk.^33^, ^34^ In addition, an ECG analysis was not performed to include specific variables (eg, P‐wave duration) in our analysis of predictors of AF occurrence.

## 5 CONCLUSIONS

In the present study, pacemaker‐detected SA showed a considerable variability during follow‐up, suggesting that continuous monitoring might be superior to a single overnight sleep study for classifying SA severity. We confirmed the association between device‐based RDI and higher risk of AF, and we also observed an association between higher RDI max values and the combined end‐point of all‐cause death or stroke among patients with no previous history of AF.

## CONFLICT OF INTEREST

Sergio Valsecchi is employee of Boston Scientific, Inc. Giuseppe Boriani reported speaker's fees of small amount from Boston, Biotronik and Medtronic. No other conflicts of interest exist.

## Data Availability

The dataset analyzed during the current study is available from the corresponding author on reasonable request.
